# The relationship between delay of gratification and work engagement: The mediating role of job satisfaction

**DOI:** 10.1016/j.heliyon.2022.e10111

**Published:** 2022-08-11

**Authors:** Yujia Ren, Rong Tang, Menglong Li

**Affiliations:** a Institute of Physical Education, Hunan First Normal University, Changsha, 410205, China; b Xiangtan Medicine & Health Vocational College, Xiangtan, Hunan, 411005, China

**Keywords:** Rural PE teacher, Education in rural, Career planning, Career development, Work enthusiasm

## Abstract

Undoubtedly, teachers remain at the forefront of implementing rural education. This indicates the significant impact their job involvement has on the overall achievements of schools in rural areas. Studies have found that teachers in rural areas often face more difficulties and fewer opportunities in their work. In general, they need a stronger ability to tolerate delay of gratification if they want to have higher job involvement. However, there is currently insufficient research on the impact of career delay of gratification on teachers' job involvement.

This study has been conducted to explore the relationship between the delay of gratification and the level of job involvement among physical education (PE) teachers in rural areas, as well as the mediating effect of job satisfaction. 530 PE teachers in the rural areas of Hunan Province, China, have been selected through random sampling as the participants of this study. They have been required to anonymously fill in a Delay of Gratification Scale, Job Involvement Scale and Job Satisfaction Scale to collect the data for investigation and analysis. The study found positive correlations among delay of gratification, job satisfaction, and job involvement among PE teachers in rural areas (P < 0.01), with job satisfaction playing a partial mediating role and intervening variable in the relationship between delay of gratification and job involvement (P < 0.01). Ultimately, delays in gratification directly and, through job satisfaction, indirectly promotes the job involvement of the teachers. The findings of this study reveal the impacts of delaying gratification on the job involvement of PE teachers in rural areas and provide a theoretical basis for increasing the job involvement of PE teachers in rural areas.

## 1 Introduction

Work engagement is defined as an individual's positive attitude towards work, manifested as positive qualities such as optimism, creativity, and engagement (Lesener et al., 2020). This emphasizes effective work engagement as the important premise of ensuring the teaching quality that, not only directly influence teachers' career development and students' learning, but also has a far-reaching influence on the development of education in the country. On January 20, 2018, it was mentioned in the Opinions of the CPC Central Committee and The State Council on Comprehensively Deepening the Reform of Construction of Teacher Group in the New Era issued by the Chinese government that “teachers' social status should be constantly improved to make them become the enviable” (Mao et al., 2018). This fully shows the government's determination to resolve a series of problems faced by teachers. Among them, improving teachers' work engagement is one of the key problems to be urgently solved.

Being widely classified as a developing country, China is also heavily agricultural, where rural education plays a crucial role in national education. Developing rural education and rural schools to satisfy the learning needs of their respective locale is a key factor in improving the quality of workers, as part of China's sustainable development policies on education. Rural teachers refer to teachers working in primary and middle and schools in rural areas. Researchers (Wang and Liu, 2020) have found that among rural teachers, PE teachers had the most prominent problems. They had low social status and salary, their teaching ability was poor, and the work engagement of rural PE teachers was low. This strongly argues the necessity to put more concern into the work engagement of rural PE teachers.

Delay of gratification was first proposed in an experiment of Delay of Gratification designed by Walter Mischel, an American professor of Psychology, which includes the concept, experimental research paradigm, and research results of delay of gratification (Mischel, 1961). This study led to subsequent multi-field and cross-cultural studies of many researchers on delay of gratification.

Current studies on delay of gratification are gradually shifting towards studies on some non-cognitive factors from the constant summary and innovation of research experiences in the study of children's cognitive factors. Liu and Wang (2021) examined 474 employees and found that employees with strong self-control ability are more willing to engage in citizenship behaviors that are conducive to the organization and their colleagues. They also concluded that career delay of gratification and job satisfaction play significant mediating roles between the relationship between self-control and organizational citizenship behavior. Xayavongsa and Pholphirul (2019) investigated least developed countries and noticed that those who are self-employed with high tendencies of delay of gratification have better business performance, thus experience more fairness in distribution. These findings provide new insights for management on the mechanism, whereby delay of gratification promotes and maintains employees' organizational behavior. Liu et al. (2007) found that there was a significant positive correlation between employees' delay of gratification and organizational career management, career commitment, and job satisfaction.

A few studies on the delay of gratification among teachers are available as well. Mohsin and Ayub (2014) investigation of 150 high school teachers in Pakistan revealed a negative correlation between procrastination and job satisfaction and a positive correlation between delay of gratification and job satisfaction. Procrastination and delay of gratification have also been found to be significant predictors of work-related stress, which was also observed to be a significant predictor of job satisfaction. Although the application scope of study on delay of gratification has been constantly expanding, the research objects, which had extended to adolescents, adults, and special groups of people (obese children, criminals, etc.) and involved a variety of fields (Weinert et al., 2022; Feng and Zi, 2020), rural teachers in developing countries had rarely been selected as research objects which was, to some extent, detrimental to the sustainable development of education.

Based on previous studies, individuals with stronger delay of gratification have higher organizational commitment and work harder. Thus, delay of gratification is important both in the development of individuals and organizations. Accordingly, the theory of delay of gratification has been innovatively applied in this study to Chinese PE teachers in rural areas. The focus of discussion revolves on the relationship between delay of gratification and job involvement, as well as the mediating effect of job satisfaction. The study aims to provide reference for augmenting the level of job involvement among PE teachers in rural areas. The study also hopes that the results of this survey can provide a new perspective for developing countries to improve the quality of PE teachers in rural areas in order to promote the sustainable development of education, optimize education and eliminate poverty.

The next section reviews the relevant studies on the relationship between delay of gratification, job satisfaction, and job engagement, and puts forward corresponding hypotheses. Section 3 describes the methods and data used. Section 4 illustrates the results of this study. Section 5 concentrates on the analysis of these results. Section 6 provides the conclusion and research implications of this study.

## 2 Theoretical analysis and model hypothesis

### 2.1 Work engagement

Work engagement is defined as a positive and fulfilling state of mind related to work which includes three dimensions: vigor, dedication, and absorption (Xanthopoulou et al., 2009). Vigor is described as the physiological dimension of work engagement, which manifests as employee and energy at work, levels of perseverance, persistence when encountering difficulties, and willingness to exert more effort. Dedication is the cognitive dimension of work engagement, which manifests as the recognition of work and workers experiencing a high-level sense of meaning, leading to increased willingness to engage in work. Absorption is the motivation dimension of work engagement, which manifests as immersion in work (Zhang and Guo, 2017). Work engagement reflects employees' recognition of the current job and the individual's belief in the importance of the current job (Green et al., 2017). Employees with high work engagement are more positive at work, thus increasing the overall effectiveness of the organization.

### 2.2 Delay of gratification

As an important personality trait, delay of gratification is found to be closely related to work engagement (Jia et al., 2015). Under the perspective of career growth, individuals with strong tendencies of delay of gratification conduct more future-oriented actions, hoping to realize more long-term and valuable career goals, leading to increased willingness to invest more energy and time in work, which results in better work performance. Consequently, these individuals understand how increased work input leads to better chances in realizing their long-term goals. This idea promotes them to dedicate more time and effort to work. In contrast, individuals with weak tendencies of delay of gratification, who focus more on immediate gratification, will more likely exert less effort at work and slack off (Wang and Qiao, 2018), affecting individual and organizational work efficiency and work engagement.

### 2.3 Job satisfaction

The concept of job satisfaction was first proposed by Hoppock (1975), who thought that it was an employee's subjective reaction to the work situation and their major feelings to the working environment in physical and psychological aspects. According to the theory of planned behavior (Ajzen, 2020), actual behavior is influenced by behavioral intention, while behavioral intention is influenced by the interaction of behavioral attitudes, subjective norms, and perceived behavioral control. Job satisfaction is therefore described as the emotion or attitude of employees to work, which is found to have a close relationship with work engagement.

Previous studies have found that job satisfaction can positively predict work engagement (Yu et al., 2016; Orgambídez-Ramos et al., 2014; Pongton and Suntrayuth, 2019). These studies reveal the positive relationship between the job satisfaction of employees and their enthusiasm to their organization, ultimately affecting their work engagement. Firstly, this is most likely subject to social exchange theory, which states that when employees are satisfactory in all aspects of their work, they are likely to provide a higher work engagement to their organization (Wang et al., 2019). Secondly, general consensus suggests on one hand, that job satisfaction is a prerequisite of job burnout, while work engagement on the other hand emerges as the opposite of job burnout. Therefore, job satisfaction has been reckoned in this study as the prediction factor of work engagement (Li and Feng, 2017). Liu and Wang (2021) illustrate how employees' delay of gratification can predict their job satisfaction. Employees with stronger delay of gratification focus more on long-term career goals rather than short-term gain and loss, which might correlate with higher job satisfaction. In conclusion, stronger delay of gratification resulted in higher job satisfaction and increased their willingness and motivation in work engagement.

There exists strong evidence on the capacity for delayed gratification to make teachers more engaged with their work. The study therefore puts forward the following hypotheses as well:

Hypothesis 1 The tendency of delay of gratification of rural PE teachers can positively predict the level of their work involvement.

Hypothesis 2 Job satisfaction of rural PE teachers can positively predict their work engagement.

Hypothesis 3 the influence of delay of gratification of rural PE teachers on their work engagement can be measured by the mediating variable-job satisfaction.

## 3 Methods

### 3.1 Participants

It is necessary to first estimate the size of the sample before starting a survey. Some scholars (Hoogland and Boomsma, 1998) claimed that the sample size should be more than 10 times the number of questions. However, in the regression model and structural equation model, a larger sample size is likely to enhance the statistical effectiveness. Therefore, Kline (1998) suggested that the sample size should be more than 20 times the number of questions. Considering that there are 23 items in the three psychological scales adopted in this study, it has been believed that the sample size should be more than 460. From September to December 2020, a questionnaire survey was conducted through random sampling among PE teachers in rural primary and secondary schools in Hunan Province, China. 552 copies of the questionnaire have been distributed, with 530 valid copies collected and a response rate of 96.01%. Among the 530 participants, 231 (43.6%) were male and 299 (56.4%) were female.

With the consent of their Supervisors, the participants have been invited to take part in this study. They have all been informed of the purpose of this study, the nature of the questionnaire, and the use of their data strictly for research purposes only, with adherence to principles of anonymity. Those who had interest in this study have been asked to sign the consent form and fill in the questionnaire above. Whilst filling in the questionnaire, the research team physically distanced themselves from the scene so that the participants could answer at ease. It took about 10 min to complete the questionnaire. This study was also conducted after getting the consent of the Human Subject Review Board from relevant research departments.

### 3.2 Research tools

#### 3.2.1 Demographic information scale

This scale covers the gender, age, occupational titles, and educational background of each participant.

#### 3.2.2 Measurement of delay of gratification

Based on a questionnaire of delay of gratification (Ray and Najman, 1986), Liu et al. (2007), conducted an open questionnaire survey on subjects in China. The questionnaire provided the following questions, among others: “Do you often give up what you want to do in order to better complete your tasks and long-term goals at work? If so, please list them in as much detail as possible”; “Do you often postpone your jobs for something enjoyable and impulsive? If so, please list them in detail as much as possible.”

Through the survey, 112 responsive items and 8 items with good effectiveness have finally been extracted after statistical testing to form the questionnaire of delay of gratification for the study of careers. The Likert 4 points scoring method has been adopted, with the score range of 1–4 points representing ‘Very Inconsistent’ to ‘Very Consistent’ responses. The Cronbachα coefficients of the total scale and the two subscales of work delay and career delay were 0.776, 0.759 and 0.707, respectively. The scores of each item in the scale had significant correlation with the total scores, which ranged from 0.476 to 0.828, showing that the scale had good internal homogeneity. Meanwhile, the structural validity and scale validity were also Sufficient (Liu et al., 2007).

#### 3.2.3 Measurement of work engagement

The simple version of the Utrecht Work Engagement Scale, UWES-9, prepared by Schaufeli et al. (2006) has been used, which included 9 items and used the Likert 7 points scoring method: with 1 point meaning “Never” to 7 points meaning ‘Always’. A higher score meant higher work engagement. The α coefficient of the scale in the study was 0.88. Li et al. (2013) has previously carried out its verification under the context of Chinese culture, showing good reliability and validity. It shows good reliability and validity when used in the sample of Chinese teachers (Shi et al., 2021).

#### 3.2.4 Measurement of job satisfaction

The study used the job satisfaction scale prepared by Schreisheim and Tsui (1980). The scale was single dimension-structured, which included 6 items to evaluate satisfaction with one's job, one's supervisor, colleagues, income, opportunities for promotion, to describe and evaluate job satisfaction. All items in the scale have been positively scored with the Likert 5 points scoring method. Available responses ranged from ‘Strongly Agree’, ‘Agree’, ‘No Opinion’, ‘Disagree’ and ‘Strongly Disagree’ represented by 5 points to 1 point answers, respectively. A lower score meant lower job satisfaction, and vice versa. The internal consistency coefficient of the scale was 0.688 (Schreisheim and Tsui, 1980). It shows good reliability and validity when used in the sample of Chinese teachers (Li et al., 2019).

### 3.3 Statistical method

Mediating effect has been tested mainly following the latest procedure of testing mediating effects proposed by Wen and Ye (2014) and verified through a structural equation model built by AMOS21.0 software. The significance of mediating effect has been directly tested using Bootstrap, a widely accepted better method at present (Zhang et al., 2022). The difference is statistically significant at P < 0.05.

## 4 Results

### 4.1 Basic information of participants

In this study, 552 copies of the questionnaire were distributed, and 530 valid copies were collected, with a response rate of 96.01%. The details are given in Table 1.

**Table 1:** Basic information of participants.

Basic information		n	%
Gender	Male	231	43.6
	Female	299	56.4
Type of school	Primary school	217	40.9
	Junior High school	313	59.1
Age	20–30	182	34.3
	31–40	148	27.9
	41–50	125	23.6
	51–60	75	14.2
Teaching years	∼5	184	34.7
	6–10	59	11.1
	11–20	89	16.8
	21–30	124	23.4
	31–	74	14.0
Title	Nothing	82	15.5
	Primary	164	30.9
	Intermediate	207	39.1
	Senior	77	14.5
Educational level	Junior High school and below	1	0.2
	Technical Secondary school/High school	9	1.7
	College graduates	158	29.8
	Bachelor's degree	361	68.1
	Master's degree	1	0.2
Marital status	Unmarried	125	23.6
	Married	394	74.3
	Other	11	2.1
Average monthly wage income (RMB)	<2000	3	0.6
	2000–	48	9.1
	3000–	191	36.0
	4000–	139	26.2
	5000–	102	19.2
	6000–	35	6.6
	7000–	12	2.3
Total		530	100

### 4.2 The direct influence of delay of gratification and job satisfaction on work engagement

The correlation analysis of delay of gratification, job satisfaction and work engagement were carried out first, and the results have shown that there was significant positive correlation among the three aspects, where the correlation coefficient between delay of gratification and job satisfaction was 0.463, between delay of gratification and work involvement was 0.470, and between job satisfaction and work involvement was 0.515 (see Table 2).

**Table 2:** Correlation analysis of delay of gratification, job satisfaction and work engagement.

	Work delayed gratification	Career delay gratification	Delay of gratification	Job satisfaction
Career delay gratification	0.620∗∗			
delay of gratification	0.902∗∗	0.898∗∗		
job satisfaction	0.278∗∗	0.323∗∗	0.334∗∗	
work engagement	0.382∗∗	0.452∗∗	0.463∗∗	0.441∗∗

∗∗P < 0.01.

Then, work engagement was used as the dependent variable, delay of gratification and job satisfaction were used as independent variables, the multiple linear regression analysis showed that both delay of gratification and job satisfaction had independent positive correlation with work engagement, and the two independent variables – delay of gratification and job satisfaction could explain 33.1% of the variation in work engagement, as shown in Table 3.

**Table 3:** Direct influence of delay of gratification and job satisfaction on work engagement.

Model		Non-standardized coefficient		Standardization coefficient	*t*	*P*
		B	Standard error			
1	(Constant)	15.074	2.215		6.804	<0.001
	Delay of gratification	1.122	0.093	0.463	12.004	<0.001
2	(Constant)	7.016	2.294		3.058	0.002
	Delay of gratification	0.860	0.093	0.355	9.232	<0.001
	Job satisfaction	0.802	0.096	0.323	8.385	<0.001

### 4.3 Delay of gratification mediated work engagement through job satisfaction

**Figure 1:**
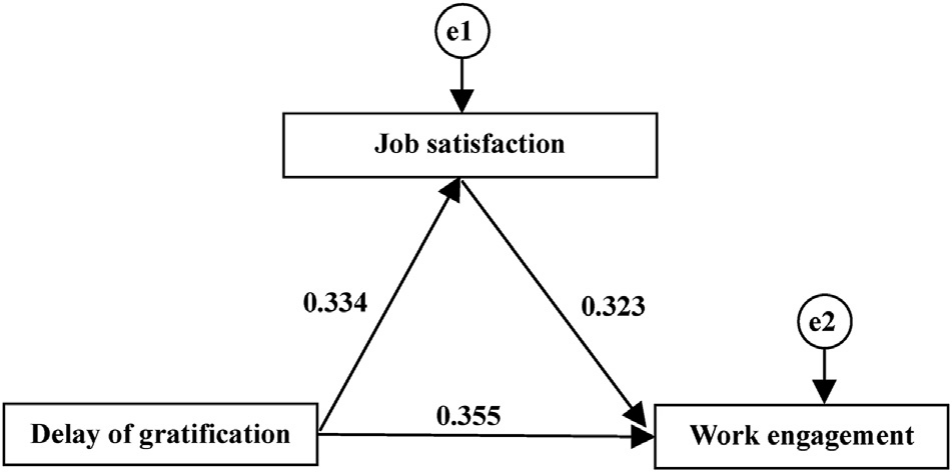
The mediating role of job satisfaction in delay of gratification and work engagement.

According to the theoretical hypothesis put forward in the preface, the model of this study was established (Figure 1), where delay of gratification was the independent variable, work engagement was the dependent variable, and job satisfaction was the intermediary variable.

The test results of three main paths of the model were shown in Table 4, where all three paths were statistically significant. Therefore, job satisfaction partially played a mediating role in the relationship between delay of gratification and work engagement.

**Table 4:** Test results parameters of main paths of the model.

Path	Non-standardized coefficient	Standard error	C.R.	P
Job satisfaction ← delay of gratification	0.325	0.040	8.153	<0.001
Work engagement ← delay of gratification	0.860	0.093	9.249	<0.001
Work engagement ← job satisfaction	0.802	0.095	8.401	<0.001

The mediating effect of the model was shown in Table 5. It could be seen that the total effect of delay of gratification on work engagement through job satisfaction was 0.463 (0.373–0.542), among which the direct effect was 0.355 (0.258–0.436), accounting for 76.7% of the total effect; the indirect effect through job satisfaction was 0.108 (0.070–0.151), accounting for 23.3%, and the above effects were statistically significant (P < 0.05).

**Table 5:** Bootstrap analysis of the mediation effect of the model.

Effect type	Effect value	95%CI	P
Direct effect	0.355	0.258–0.436	0.001
Indirect effect	0.108	0.070–0.151	0.001
Total effect	0.463	0.373–0.542	0.001

## 5 Discussion

### 5.1 The influences of delay of gratification on work engagement

The results of the study have shown that delay of gratification could strongly predict work engagement, which was consistent with previous studies (Zhou and Su, 2021). Rural PE teachers with stronger delay of gratification had more valuable career goals, higher identification with work, significantly recognized the value of their work, and were willing to put more energy and efforts in work to achieve their career goals. This signified their higher work engagement.

On the contrary, individuals with lower delay of gratification focused more on short-term benefit and rest and recreation that could immediately satisfy them rather than long-term valuable goals. They were more likely to slack off and had a negative attitude towards work, hence signifying lower work engagement (Wen and Liu, 2017).

After investigating 1176 teachers aged 20–60 in rural areas of China, Shi et al. (2021) also pointed out a significant positive correlation between delay of gratification and job involvement among teachers in poor rural areas. This coincides with the result reported in this study, further signaling a strong relationship between career delay of gratification and positive job outcomes. As mentioned earlier, delay of gratification turns individuals' attention to a more valuable career goal and enhances their ability to independently control the interference of irrelevant stimuli or events in the process of achieving this goal (Mohsin and Ayub, 2014). Delay of gratification, on one hand, can help individuals assuage their existing resources when they are consumed. On the other hand, it can also play the function of preserving and expanding resources so that individuals can better respond to work requirements and improve their job involvement (Shi et al., 2021). This result has important implications for practice and policy; it suggests that school leaders should pay more attention to and focus on improving career delay of gratification when engaging PE teachers in rural areas in vocational training.

Consequently, PE teachers are more likely to clarify their future development prospects and the significance of their career, thus having a higher level of job involvement. For example, daily teacher training should not be limited to the training of professional teaching skills but should also cover some training for improving the comprehensive literacy of rural areas, including in areas such as career planning. Leaders can also learn more about teachers' goals and expectations for their future career development and give them as many opportunities as possible to help them achieve some of their own goals in the school. Meanwhile, they are supposed to appropriately assist teachers to formulate long-term career plans and guide them on how to gradually approach their long-term goals based on their immediate work, so as to reinforce their awareness of career delay of gratification.

### 5.2 The influences of job satisfaction on work engagement

As mentioned, the job satisfaction of rural PE teachers could positively predict their work engagement, which is consistent with studies using other careers as research objects such as enterprise employees (Chen et al., 2021), doctors (Gu et al., 2021), primary and secondary school teachers (Pepe et al., 2021), and preschool teachers (Wang, 2017). This indicates that the higher the job satisfaction rural PE teachers had, the more willingness was evident in their engagement in PE teaching, and the more enthusiasm was exhibited in their work. This could be explained by two things: Firstly, rural PE teachers with higher job satisfaction were more likely to find the value of themselves and their interests at work, and were more likely to sense the positive factors in their work than teachers with lower job satisfaction, thus having higher work engagement (Li et al., 2019). Secondly, based on social exchange theory and reciprocal rules, rural PE teachers would perform better if they felt recognized, respected, and cared for by schools (Shi et al., 2021). Therefore, when PE teachers were satisfied in all aspects of the school, they would have higher work engagement and improved the overall efficiency of the school.

This has indicated that comparing to delay of gratification, improving job satisfaction of rural PE teachers might be a better plan of action to improve their work engagement. As observed by Carson et al. (2016), “the kids and control”, “our administration and marginalization”, and “the relationship with my fellow coworkers” are the main factors influencing PE teachers' job satisfaction. Hence, it is recommended that administrators remain attentive to teachers' need for autonomy, promote school and departmental cultures centered on collegiality and community, and help teachers retain enjoyment to work with children (Carson et al., 2016).

Wang and Liu. (2020) also pointed out that PE teachers in rural areas are now featured with seriously insufficient job satisfaction due to a variety of reasons. Firstly, PE teachers in rural areas cannot freely arrange their time under heavy tasks. Primary schools in rural areas are short of teachers due to salary and geographical constraints, but they are still required to offer various courses under the requirements of ensuring daily teaching, fulfilling the provisions of the school syllabus and promoting students' all-round development. Moreover, some courses for which corresponding teachers are not available can only be taught by those teachers in charge for other courses. In some cases, PE teachers with less daily class hours also served as teachers of other courses in rural primary schools.

Secondly, the actual sports venues and sports equipment in rural areas basically fail to meet the teaching requirements of the PE curriculum. The acquisition of sports skills is inseparable from the assistance of well-established sports venues and good equipment (Meng and Wang, 2022). Nevertheless, rural primary schools have not met the actual needs for venues, equipment, and facilities. This results in more obstacles for PE teachers to teach in rural primary schools. Consequences for this lack of facilities include poor teaching effect, decline in teachers' job satisfaction, and reduction in their job involvement.

This also highlights the necessity to provide PE teachers in rural areas with more humanistic care (Meng and Wang, 2022). For example, work tasks should be assigned reasonably according to teachers' personal conditions. School leaders should have timely and constant communication with teachers, understand their job attitudes and needs, and arrange their daily work in a way that allows them to better control their own time. At the same time, leaders are also suggested to increase the investment of funds in sports and create favorable conditions for PE.

The teaching of sports is closely related to the teaching level of teachers and the completeness of necessary venues and equipment. In this regard, it is suggested to increase financial investment in teaching facilities of PE in rural primary schools, equip rural primary schools with sufficient sports equipment, meet students' needs for physical exercise, and change the imbalance between more users and fewer facilities. In addition, social individuals or social public welfare organizations can also be encouraged to donate equipment and funds for rural primary schools which would greatly improve PE in rural primary schools.

### 5.3 The mediating role of job satisfaction in delay of gratification and work engagement

From the test results above, delay of gratification can significantly predict the job involvement of PE teachers in rural areas, and job satisfaction can positively predict their job involvement. This result is basically consistent with the result obtained by Li et al. (2019) through the investigation and analysis of 309 college PE teachers in China who filled in Professional Identity Scale, Delay of Gratification Scale and Satisfaction Index Scale anonymously.

To further identify whether job satisfaction plays a mediating role between professional delay and job involvement among PE teachers in rural areas, a mediating model has been constructed through AMOS software to test the possible mediating effect of job satisfaction. The test results show that job satisfaction plays a partial mediating role in the relationship between delay of gratification and job involvement among PE teachers in rural areas; delay of gratification not only directly predicts the job involvement of PE teachers in rural areas, but also predicts their job involvement through job satisfaction.

The reason for this might lie in the following aspects: Firstly, rural PE teachers with stronger willingness of delay of gratification often had better career management, were more focused more on long-term goals, and seldom consider temporary gains and losses or ups and downs at work. They had higher job satisfaction and were therefore more willing to put more time, energy, and effort into work, which resulted in better performance and outcome (Tao, 2018). Secondly, rural PE teachers with stronger delay of gratification had better performance in overcoming difficulties, adapting to social environments, professional self-development, self-realization, etc., which were all positively correlated to job satisfaction (Zhang, 2017). Higher job satisfaction further enhanced their willingness and motivation in improving their work engagement. Therefore, delay of gratification can improve work engagement through job satisfaction.

The results of this study have important implications for the research and practice of rural education. Findings on how job satisfaction play an important role in promoting career delay of gratification and job involvement among PE teachers in rural areas enables relevant researchers and policymakers to have a more comprehensive understanding of the performance evaluation of PE teachers in rural areas. It has been revealed in previous studies that full support from leaders, high performance standards, and well-established cooperative relations among colleagues can improve the satisfaction of PE teachers (Eirín-Nemiña et al., 2022), while administration and marginalization would significantly reduce the job satisfaction of PE teachers (Carson et al., 2016).

In this sense, administrators are recommended to increase the care for PE teachers in rural areas by keeping trace of their needs for autonomy, enlarge the existing population of PE teachers in rural areas, and enrich PE teaching facilities in rural schools. In addition, the results regarding delay of gratification in this study also yield other implications: those who can resist temptation temporarily, that is, those who have the tendency of delay of gratification, are more likely to achieve success in career development (Shi et al., 2021). At the same time, the results of this study also show that delay of gratification can improve the sense of job involvement among PE teachers in rural areas.

Accordingly, from the perspective of school administrations, it is wise to select those employees with strong ability to tolerate delay of gratification in the recruitment stage and create conditions to reinforce their ability to tolerate delay of gratification. These efforts are important in reducing the turnover of teachers in rural areas and in the long-term development of rural PE. Teachers themselves learn to choose and complete work with tolerance to delay of gratification, thus having higher job satisfaction and long-term occupational competitiveness.

## 6 Conclusion and research implications

### 6.1 Conclusion

This study has been conducted to discuss the relationship between delay of gratification and job involvement among PE teachers in rural areas and analyze the mediating role of job satisfaction between them. The results show 4 salient points: 1) There is a significant positive correlation between delay of gratification and job involvement among PE teachers in rural areas. 2) There is also a significant positive correlation between job satisfaction and job involvement. 3) A significant positive correlation is also found between delay of gratification and the teachers' job satisfaction. Lastly, 4) Job satisfaction plays a mediating role in the relationship between delay of gratification and job involvement.

### 6.2 Implications

The results of this study have important implications for the research and practice of rural education. The findings presented enables relevant researchers and policymakers to have a more comprehensive understanding of the performance evaluation of PE teachers in rural areas. Specifically, delay of gratification not only directly promotes the job involvement of PE teachers in rural areas, but also indirectly promotes their job involvement through job satisfaction. The results of this study further enrich relevant research on the relationships among delay of gratification, job satisfaction and job involvement. Specifically, they signal the possibility of augmenting the job involvement level of PE teachers in rural areas by improving their job satisfaction. This is especially valuable considering how at present, there are limited existing studies in this regard.

### 6.3 Limitations and future directions

Firstly, only the mediating effect of job satisfaction between delay of gratification and job involvement has been examined in this study. Detailed analysis of the mediating effect of delay of gratification and job involvement in each dimension has not been fully dissected and illustrated, along with the exploration of possible regulation mechanisms between these three variables. Secondly, the sample size will be expanded in future research, and an attempt will be made to investigate teachers responsible for the teaching of different disciplines in rural areas. Ultimately, the further in-depth and comprehensive investigation of these three variables, as well as their deeper relationships in future research, will contribute to the development of rural education and provide reference for educational policies in developing countries.

### Compliance with ethical standards

All procedures performed in studies involving human participants were in accordance with the ethical standards of the institutional and/or national research committee and with the 1964 Helsinki declaration and its later amendments or comparable ethical. Informed consent was obtained from all individual participants included in the study.

## Declarations

### Author contribution statement

Menglong Li: Conceived and designed the experiments; Analyzed and interpreted the data; Contributed reagents, materials, analysis tools or data; Wrote the paper.

Yujia Ren: Conceived and designed the experiments; Performed the experiments; Analyzed and interpreted the data; Contributed reagents, materials, analysis tools or data; Wrote the paper.

Rong Tang: Performed the experiments; Contributed reagents, materials, analysis tools or data.

### Declaration of interests

The authors declare no conflict of interest.

### Data availability statement

Data will be made available on request.

### Funding statement

This study was supported by general program of Humanities and Social Sciences Research of the Ministry of Education (18YJC890030)

### Additional information

No additional information is available for this paper.
